# Role of Raf-1 Kinase in Diabetes-Induced Accelerated Apoptosis of Retinal Capillary Cells

**Published:** 2008-03

**Authors:** Steven P. Rayappa, Renu A. Kowluru

**Affiliations:** *Department of Ophthalmology, Kresge Eye Institute, USA*

**Keywords:** apoptosis, diabetic retinopathy, endothelial cells, Raf-1, small molecular weight G-protein

## Abstract

Small molecular weight G-proteins serve as fundamental signaling switches that regulate cell fates by coupling receptor activation to downstream effector pathways. H-Ras, a small molecular weight G-protein, in its active form, recruits Raf. Activated Raf via a signaling transduction pathway regulates apoptosis. Our previous studies have shown that H-Ras has an important role in the loss of retinal capillary cells in diabetes. The purpose of this study is to investigate the role of Raf-1 in the development of diabetic retinopathy. Bovine retinal endothelial cells were incubated in 5 mM or 20 mM glucose in the presence of Raf-1 kinase inhibitor (10μM of GW5074), activator (200μM of ZM336374) or mitogen activated protein kinase inhibitor (30μM of PD098059) for five days. Apoptosis of endothelial cells was analyzed by ELISA and activation of Raf-1 and its downstream signaling proteins by determining genes and protein expressions. Inhibition of Raf-1 kinase repressed glucose-induced apoptosis of the cells by 75%, and this was accompanied by attenuation of activation of MAP kinase, ERK-1, nuclear transcriptional factor and caspase-3. In contrast, ZM336374 further increased glucose-induced apoptosis by 50%, and activated the signaling molecules and caspase 3 by over 30%. Further, PD098059 alone also attenuated glucose-induced apoptosis of retinal endothelial cells. These findings demonstrate that accelerated loss of retinal capillary cells in diabetes is mediated via Raf-1 kinase activation. Modulation of Raf-1 kinase activity could, in part, regulate apoptosis of retinal endothelial cells, which may ultimately contribute to the development of diabetic retinopathy.

## INTRODUCTION

Retinopathy, a blinding complication of diabetes, is a multi-factorial disease. Modern experimental techniques have identified many biochemical pathways in its development, but due to the complexity associated with its development, the exact abnormality responsible for its development remains to be identified. Consequently, targets for therapeutic interventions have been hindered.

In the development of diabetic retinopathy, accelerated apoptosis of pericytes and endothelial cells is observed before histopathological changes can be seen in the retinal vasculature (1, 2). Animal studies have shown that apoptosis of capillary cells can serve as a surrogate endpoint to screen efficacy of interventions for inhibiting the development of diabetic microvascular complications (2). Capillary cell apoptosis may not be the only process culminating in retinopathy because other cells, including neuronal cells of the retina also undergo accelerated apoptosis (3); however, their role in the manifestation of retinopathy is not clear.

Ras proteins, small molecular weight G-proteins, serve as fundamental signaling switches that regulate cell fates by coupling receptor activation to downstream effector pathways. Our studies have shown that H-Ras-mediated pathway plays a significant role in accelerated retinal capillary cell apoptosis in diabetes, which can be prevented by Ras farnesylation inhibitors and genetic manipulation of functionally active H-Ras (4, 5). Activated Ras (GTP-Ras) recruits Raf, its principal effector protein, to the plasma membrane and directly interacts with it. Activated Raf phosphorylates mitogen-activated protein kinase (MEK), which in turn phosphorylates and activates extracellular signal-regulated kinases (ERK) (6). Inhibitors of Raf kinase have shown promising results in experimental and clinical cancer therapy (7), however, their effect on the pathogenesis of diabetic complications remains to be elucidated. The purpose of this study is to investigate the role of Raf-1 in the development of diabetic retinopathy-by modulating the effect of Raf-1 kinase (inhibition and activation) on glucose-induced accelerated apoptosis of retinal endothelial cells.

## METHODS

### Retinal endothelial cells

Endothelial cells were prepared from bovine retina and cultured in Dulbecco’s Modified Eagle Medium (DMEM) containing 15% fetal calf serum (heat inactivated), 5% Nu-serum, heparin (50 μg/ml), endothelial growth supplement (25 μg/ml) and antibiotic/antimycotic in 95% O_2_ and 5% CO_2_ . The cells from 4-7^th^ passage were incubated in culture medium (DMEM containing 2% heat inactivated fetal calf serum, 10% Nu-serum, heparin, 2.5 μg/ml endothelial growth supplement and antibiotic/antimycotic) with or without 20 mM glucose. Raf-1 kinase inhibitor GW5074 ([5-Iodo-3-[(3,5-dibromo-4-hydroxyphenyl) methylene]-2-indolinone]; Biomol International, Plymouth Meeting, PA) at 0.5-50 μM, or Raf-1 kinase activating compound, ZM336372 (3-(dimethylamino)-N-[3-[(4-hydroxybenzoyl)amino]-4-methylphenyl]-benzamide) obtained from Cayman Chemical, Ann Arbor, MI) at 100-300 μM were added at the time of initiation of the experiment (8, 9). Our initial experiments demonstrated that the optimal concentration for obtaining an effect without compromising cell viability was 10 μM for GW5074 and 200 μM for ZM336372.

To specifically investigate the effect of MEK pathway in accelerated apoptosis of endothelial cells, we incubated the cells with or without MEK inhibitor PD098059 (2’-amino-3’methoxyflavone, United States Biological, Swampscott, MA) at a final concentration of 30 μM (10). Where necessary, dimethyl sulfoxide (DMSO) was used to dissolve these compounds, and the final concentration of DMSO in the incubation medium was kept below 0.01%. Appropriate controls, including DMSO control and osmotic control (20 mM mannitol) were included in each experiment. At the end of the incubation, the cells were washed with PBS, and used for protein or RNA isolation. RNA was isolated using Trizol reagent by the method routinely used in our laboratory (11), and its content and purity were confirmed by analyzing absorbance ratios at 260:280 nm. Gene expression H-Ras and ERK-1 was determined by semi- quantitative Reverse Transcription-Polymerase Chain Reaction (RTPCR), and that of Raf-1, p38 and NF-kB by real-time quantitative RTPCR (Q-RTPCR) using the templates given in Table I. Protein expression of signaling molecules were determined by western blot.

**Table 1:** Forward primer, reverse primer and Taqman probe sequences (5’→3’) of the target genes

Gene	GenBank Accession No.	Forward primer	Reverse primer

Raf-1	NM_001102505	TCGGATTCAAAGACACGGTGTTT	GCTGATAGCCAAATTGCTGAACT
p38	XM_614274.3	GCGGTTACTTAAACATATGAAGCATGAA	GCACATCATTGAATTCCTCCAGAGA
NF-*k*B	BC133594	CTCACCCTCACGAGCTAGTG	GGGCAGAGCTCAGCCT

Taqman primers and probe for target genes were designed by use of File Builder 3.0 (ABI) and synthesized by ABI. Nucleotide sequences used for primer and probe design were obtained from the GenBank database. When a bovine sequence (p38 and NF-kB) was not available, bovine expressed sequence tag sequences corresponding to the target gene were used on the basis of similarity to the corresponding human sequence. TaqMan Assays on Demand (ABI) was used for 18S (housekeeping gene); the GenBank accession number for this assay is X03205 and the amplicon length is 187 bp.

### Gene Expression

RNA was converted to cDNA using the High capacity cDNA reverse transcription kit with an RNase inhibitor. Primers for the target genes were designed using Applied Biosystems software Primer Express 3.0 and synthesized by Integrated DNA Technologies (Coralville, IA). For semi- quantitative RTPCR, 0.5 μg of cDNA template was added to 100 pmol of forward and reverse primers (H-Ras: Fwd 5’-CCCACCCTGCCCAAGAG-3’, Rev 5’-TTGACGTGGTTGATAGCAAACAC-3’, and ERK: FWD 5’-TGGAGGTGTGGTGTTCAAGGT-3’, Rev 5’-GCTCCATGCAGATGCTGATCT-3’) and 1 unit of GoTaq DNA Polymerase (Promega, Madison, WI). Following the PCR amplification, the DNA was run on a 1.2% agarose gel at 80 V. DNA ladder, 100 bp, was simultaneously run on each gel. The bands were visualized with the UVP Bio-Doc it Imaging System (UVP LLC., Upland, CA), and band intensities were quantified by Un-Scan-It Gel digitizing software.

Q-RTPCR, reactions were carried out using a total of 300 ng cDNA templates in 96 well plates using the ABI-7500 sequence detection system (11). Each sample was analyzed in triplicate. Data were normalized to the expression of 18S in each sample and the fold change in gene expression relative to normal was calculated using the ddCT method. The GenBank accession numbers and the amplicon length for the target and housekeeping genes used are provided in Table 1.

### Western blotting

The cells were sonicated in a lysis buffer containing 30 mM Tris-HCl, pH7.5; 10 mM ethylene glycol tetra acetic acid, 5 mM ethylene diamine tetra acetic acid, 250 mM sucrose, 1% Triton X-100, 1 mM sodium fluoride, 1 mM sodium vanadate, 1 mM phenylmethylsulfonyl fluoride and protease inhibitors cocktail (aprotinin, leupeptin and pepstatin). The homogenate was centrifuged at 900 xg for 5 minutes to remove cellular debris. Protein concentration was estimated in the supernatant by bicinchoninic acid assay (Sigma-Aldrich, St Louis, MO) using bovine serum albumin as the standard. Protein (20-30 μg) was separated by SDS-PAGE on a 4-16% gradient gel and blotted to nitrocellulose membrane. The membranes were blocked in 5% nonfat milk suspended in Tris-buffered saline containing 0.02% Tween 20. The membranes were incubated with the target primary antibody (H-Ras, Raf-1 and caspase-3 from Santa Cruz Biotechnology, Santa Cruz, CA; phospho-p38 from Cell Signaling Technology, Beverly, MA) for one hour at room temperature. The blots were washed and incubated with appropriate horseradish peroxidase-coupled secondary antibody for one hour. After washing the membranes, the target proteins were enhanced by ECL reagent (Santa Cruz Biotechnology), and determined by autoradiography. The membranes were stripped and re-probed with the house-keeping protein, β-actin (Sigma-Aldrich). Each band was quantified using Un-Scan-It Gel digitizing software (Silk Scientific Inc, Orem, UT), and the protein expression levels were then quantified relative to β-actin in the same sample.

### Apoptosis

Apoptosis was determined by an ELISA method, and confirmed by measuring activation of apoptosis execution enzyme caspase-3 as follows.

Cell Death Detection ELISA^PLUS^ (Roche Diagnostics, Indianapolis, IN) that uses the monoclonal antibodies directed against DNA and histones respectively was used to quantify the relative amounts of mono- and oligo- nucleosomes generated from apoptotic cells. The cytoplasmic fraction of the cells was transferred onto a streptavidin-coated microtiter plate and incubated for 2 hours at room temperature with a mixture of peroxidase-conjugated anti-DNA and biotin-labeled anti-histone. The plate was washed, incubated with the 2,2’-Azino-di- [3-ethylbenzthiazoline sulfonate] diammonium salt (ABTS, Roche Diagnostics), and absorbance was measured at 405 nm. After separation of the cytoplasmic fraction, the nuclear pellet was suspended in 50 mM sodium phosphate buffer (pH7.5) containing 2 mM NaCl, 0.05 mM Na_2_HPO_4_ (pH 7.5) and sonicated. DNA was measured in this fraction, and apoptosis was normalized to the amount of DNA. This method is routinely used in our laboratory (4, 13).

Enzyme activity of caspase-3 was assayed in the cells by measuring the cleavage of Ac-DEVD-pNA (Biomol International) at 37°C. The absorbance of p-nitroanilide formed by the enzyme reaction was determined at 405 nm. Each sample was analyzed in duplicate. Activation of caspase-3 results in increased expression of its 17 kD active subunit. Western blotting was performed to determine the expression of this 17 kD subunit of caspase-3 (4, 13).

### Nitrite levels

Nitrite (NO) production was quantified by Greiss reagent (4). The absorbance was measured at 540 nm, and the nitrite concentration was calculated using sodium nitrite as a standard.

### Statistical analysis

Values are reported as mean ± SD. The experimental groups were compared using the nonparametric Kruskal-Wallis test followed by Mann-Whitney test.

## RESULTS

**Figure 1:**
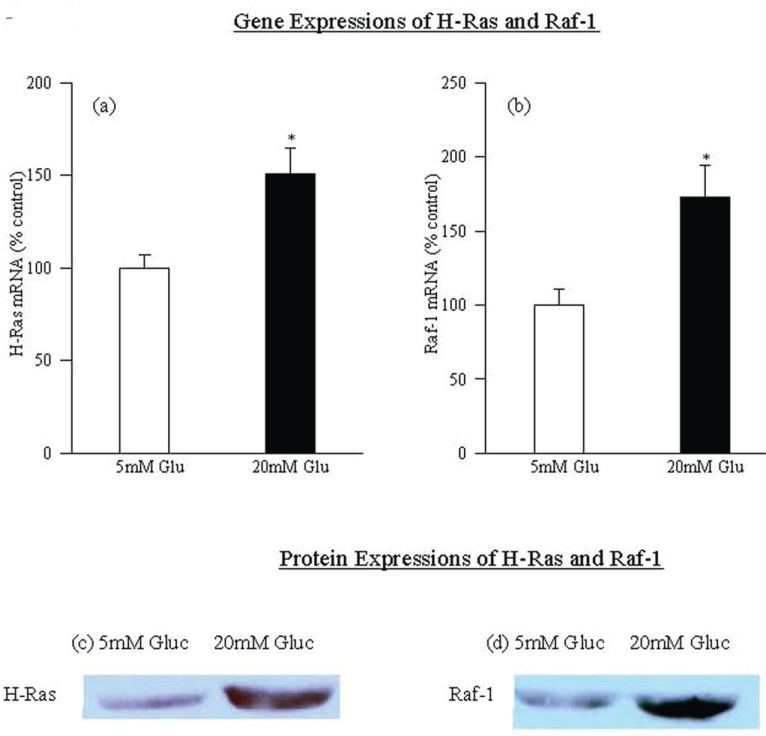
High glucose activates H-Ras and Raf-1in retinal endothelial cells. H-Ras and Raf-1 (a, b) mRNA levels were measured in endothelial cells incubated in 5 mM and 20 mM glucose medium for five days by RTPCR and Q-RTPCR respectively, using the primers described in the method section. RTPCR vales are represented as mean ± SD of the PCR product band intensity viewed on 1.2% agarose gels, and Q-RTPCR vales were normalized to that of the housekeeping gene, 18S, in the same sample. Each measurement was performed in duplicate using three (or more) cell preparations. The values obtained at 5mM glucose are considered 100%. 5 mMGlu, 5 mM glucose; 20 mMGlu, 20 mM glucose. ^*^P<0.05 compared to 5 mM glucose. (c, d) Western blots were used to confirm the effect of glucose on these two proteins; the blots presented in the figure are representatives of 4-5 experiments.

High glucose activates H-Ras and its principal effector protein Raf-1. Incubation of retinal endothelial cells with high glucose (20 mM) increased the mRNA expression of H-Ras by 50 %. In the same cells, the mRNA expression of the protein that activated H-Ras which binds to Raf-1 was elevated by over 75% (Figure 1a and b). Increases in mRNA expression of both H-Ras and Raf-1 were accompanied by parallel increases in their protein expressions (Figure 1c). Protein expression of H-Ras was increased by 45% (Figure 1c), and that of Raf-1 by about 100% (Figure 1d) in the cells incubated in 20 mM glucose compared to the cells incubated in 5 mM glucose.

**Figure 2:**
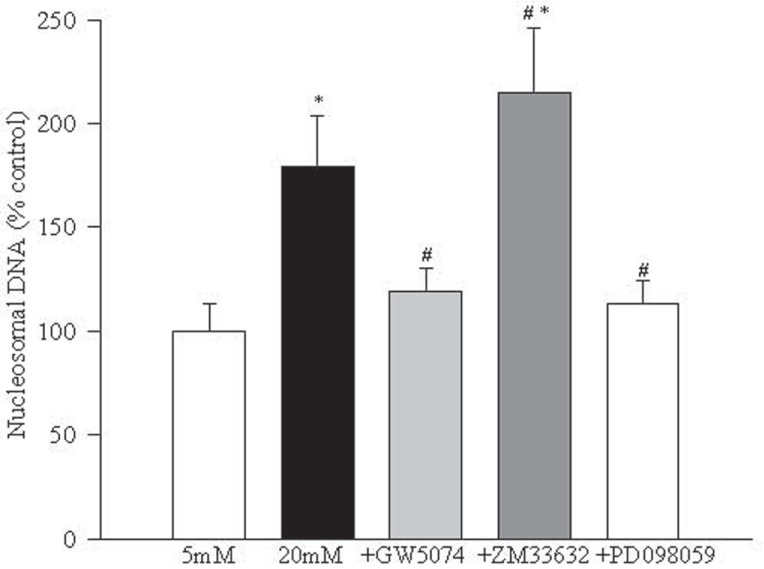
Raf-1 kinase modulates glucose-induced apoptosis of retinal endothelial cells. Apoptosis was measured by performing ELISA for cytoplasmic histone-associated-DNA-fragments using an assay kit from Roche Diagnostics. The figure shows the values obtained from the cells incubated with glucose for five days, and these values were adjusted to the total DNA. The values obtained with 5 mM glucose were considered 100%. 5 MmGlu, 5 mM glucose; 20 mMGlu, 20 mM glucose; +GW5074=20 mM glucose+10 μM GW5074; +ZM336374=20 mM glucose+200 μM ZM336374; +PD098059=20 mM glucose+30 μM PD098059. ^*^P<0.05 compared to 5 mM glucose and ^#^*P*<0.05 compared to 20 mM glucose.

Raf-1 kinase modulators regulate glucose-induced increased apoptosis of endothelial cells. Incubation of retinal endothelial cells in 20 mM glucose for 96 hours increased their apoptosis by 80% as determined by ELISA (Figure 2). The addition of the Raf-1 kinase inhibitor-GW5074 during the time of incubation with 20 mM glucose inhibited apoptosis of the endothelial cells by over 75%. To further confirm the role of Raf-1, an activator of Raf-1 kinase, ZM336372, was used. The addition of ZM336374 resulted in 50% additional increase in the apoptosis compared to those cells incubated in 20 mM glucose alone. However, when GW5074 or ZM336372 were supplemented in 5 mM glucose medium, there was no significant effect of these compounds on the apoptosis of retinal endothelial cells.

**Figure 3:**
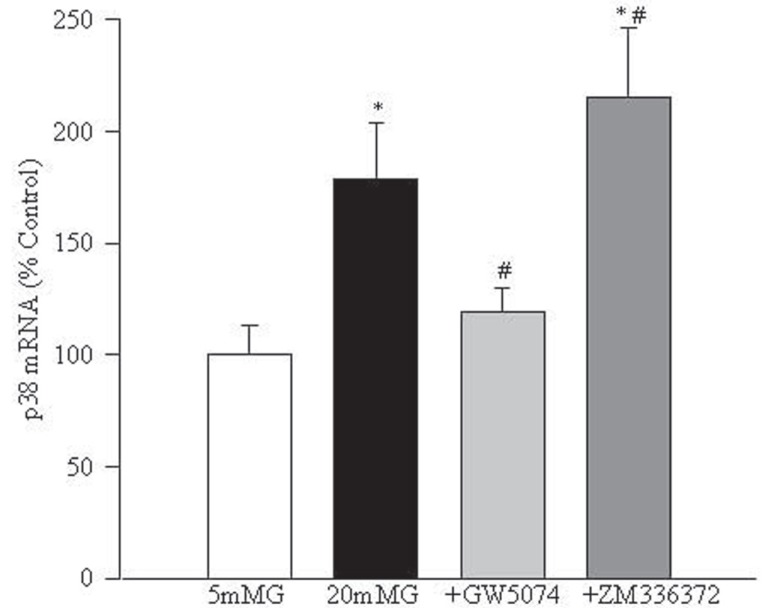
Glucose-induced increase in MAP kinase is regulated by Raf-1 kinase. Transcript expression of p38 MAP kinase was determined by Q-RT-PCR in the RNA obtained from the cells incubated in 20 mM glucose with and without modulators of Raf-1 kinase. Measurements were made in duplicate using four different cell preparations, and 100% values are the ones obtained from 5 mM glucose. 5 mMGlu, 5 mM glucose; 20 mMGlu, 20 mM glucose; +GW5074=20 mM glucose+10 μM GW5074; +ZM336374=20 mM glucose+200 μM ZM336374. ^*^P<0.05 compared to 5 mM glucose and ^#^*P*<0.05 compared to 20 mM glucose.

Raf-1 kinase modulates down-stream signaling proteins. Figure 3 shows that glucose increases the mRNA expression of p38MAP kinase in retinal endothelial cells that can be inhibited by the Raf-1 kinase inhibitor GW5074. Supplementation with ZM336372 in 20 mM glucose medium further significantly increased the gene expression of p38MAP kinase. This was confirmed by evaluating the protein expression of p-p38 MAP kinase, which showed that GW5074 significantly decreased glucose-induced p-p38MAP kinase expression (data not shown).

**Figure 4:**
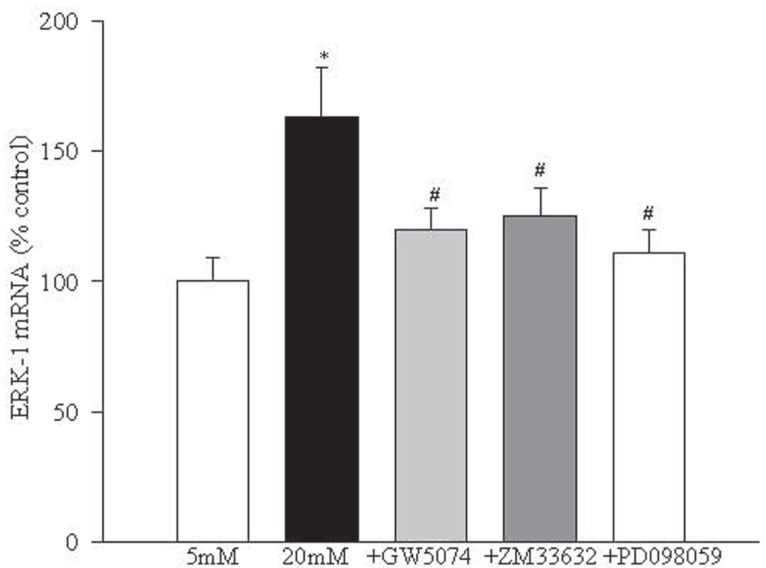
ERK-1 activation is regulated by Raf-1 and MEK. Gene transcripts of ERK-1 were measured in endothelial cells incubated in 5 mM and 20 mM glucose medium for five days by RTPCR using the primers described in the method section. The values are represented as mean ± SD of the PCR product band intensity viewed on 1.2% agarose gels. ^*^P<0.05 compared to 5 mM glucose and ^#^*P*<0.05 compared to 20 mM glucose.

ERK-1 expression, as determined by its transcript, was increased by 55-70% in retinal endothelial cells incubated in 20 mM glucose medium compared to the cells incubated in 5 mM glucose. Inclusion of GW5074 significantly decreased glucose-induced activation of ERK-1, but ZM336372 failed to produce any additional increase in glucose-induced activation of ERK-1 (Figure 4).

**Figure 5:**
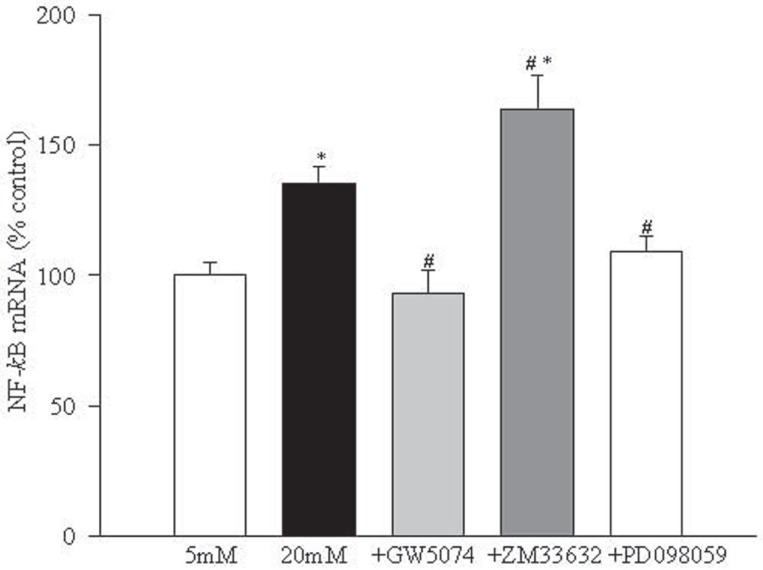
Raf-1 kinase and MEK regulate NF-kB activation: Gene expression of NF-*κ*B was determined by Q-RT-PCR using Taqman primers and probe designed by the use of File Builder 3.0 (ABI). The results are mean ± SD of three of more experiments with each measurement made in triplicate. 5 mMGlu, 5 mM glucose; 20 mMGlu, 20 mM glucose; +GW5074=20 mM glucose+10 μM GW5074; +ZM336374=20 mM glucose+200 μM ZM336374; +PD098059=20 mM glucose+30 μM PD098059. ^*^P<0.05 compared to 5 mM glucose and ^#^*P*<0.05 compared to 20 mM glucose.

Raf-1 mediated increase in endothelial cell apoptosis in hyperglycemia is via activation of NF-kB. High glucose increased the gene expression of NF-*k*B in the retinal endothelial cells by 30% compared to the cells in 5 mM glucose. Addition of Raf-1 kinase inhibitor GW5074 blocked glucose-induced increase in NF-*k*B expression while ZM336372 further increased NF-*k*B activation by 80% (Figure 5).

**Figure 6:**
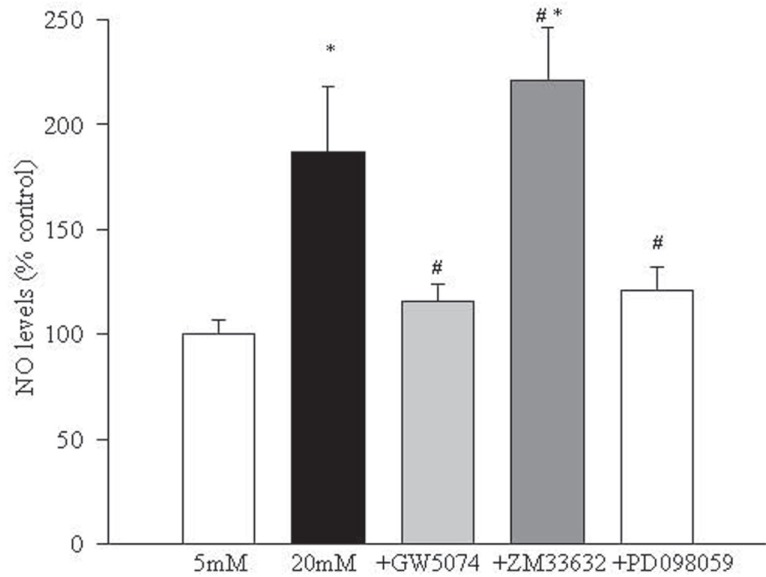
Regulation of Raf-1 kinase modulates glucose-induced increase in NO levels. NO content was measured by Greiss reagent in the cells incubated in 5 mM or 20 mM glucose medium for 5 days containing inhibitors or activator as described in the method section. Each measurement was made in duplicate using 4-5 endothelial cell preparations. ^*^ *P*<0.05 compared to 5 mM glucose and ^#^*P*<0.05 compared to 20 mM glucose.

Increase in NO concentration in endothelial cells in high glucose is inhibited by Raf-1 kinase inhibitors. As shown in Figure 6, addition of Raf-1 kinase inhibitor GW5074 prevented glucose-induced increase in NO levels in the endothelial cells. Inclusion of ZM336372 in the medium further increased NO levels in the endothelial cells by 30% compared to the cells incubated in 20 mM glucose alone. However, when ZM336372 was added in 5 mM glucose medium, the elevation in NO levels was less than 10% compared to the cells incubated without the activator (data not shown).

**Figure 7:**
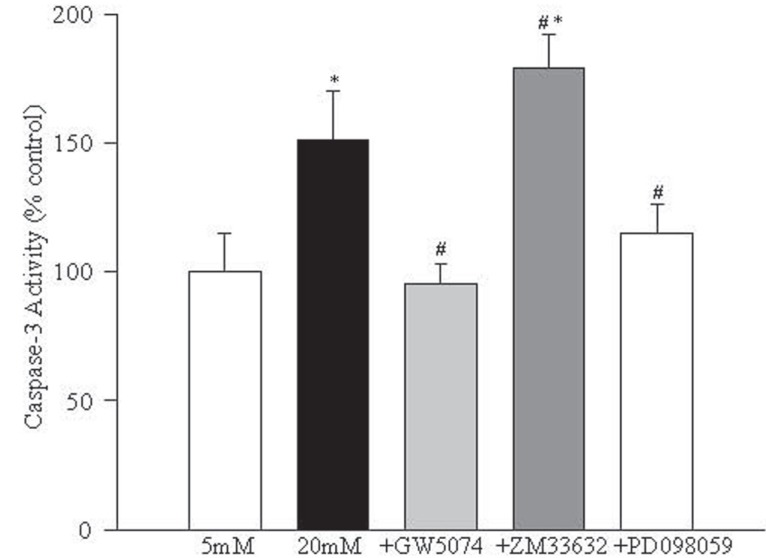
Caspase-3 is implicated in Raf-1 mediated glucose-induced increased apoptosis of retinal endothelial cells. Activity of apoptosis execution enzyme- caspase-3 was determined in the cells by measuring the cleavage of Ac-DEVD-pNA at 405 nm. The values obtained from the cells incubated in 5 mM glucose are considered 100%. Each experiment was repeated with 3-4 cell preparations, and measurements done in duplicate. 5 mMGlu, 5 mM glucose; 20 mMGlu, 20 mM glucose; +GW5074=20 mM glucose+10 μM GW5074; +ZM336374=20 mM glucose+200 μM ZM336374; +PD098059=20 mM glucose+30 μM PD098059. ^*^P<0.05 compared to 5 mM glucose and ^#^*P*<0.05 compared to 20 mM glucose.

Raf-mediated apoptosis of retinal capillary cells in high glucose is via activation of caspase-3. In order to investigate the involvement of caspase-3 activation in Raf-mediated apoptosis of retinal endothelial cells in high glucose conditions, the enzyme activity and cleavage of 17kD subunit of caspase-3 were determined. As shown in Figure 7 addition of GW5074 significantly decreased glucose-induced increase in caspase-3 activity, but ZM33632 further increased caspase-3 activity by 40% compared to the cells incubated in 20 mM glucose alone. In the same cell preparations, GW5074 significantly attenuated glucose-induced increase in the expression of 17kD subunit of caspase-3, and ZM33632 further increased its expression (data not shown).

Inhibition of MEK activation prevents increased NF-kB activation and apoptosis of endothelial cells in hyperglycemia. In order to understand the role of MEK, a down-stream signaling step of Raf-1 activation, in glucose-induced apoptosis of retinal endothelial cells, we investigated the effect of a specific and noncompetitive inhibitor of MEK phosphorylation, PD098059, on glucose-induced activation of NF-*k*B, caspase-3 and apoptosis. Supplementation with PD098059 attenuated glucose-induced increased apoptosis of retinal endothelial cells by 80% (Figure 2), inhibited activation of ERK-1 and NF-*k*B gene expressions and caspase-3 activity by over 60%, and decreased NO levels by over 70% (Figures 4-7).

## DISCUSSION

This is the first report to demonstrate that activation of Raf-1 kinase is implicated in the accelerated loss of retinal capillary cells that occurs in response to elevated glucose. The data clearly shows that pharmacological inhibition of Raf-1 kinase blocks glucose-induced increased retinal capillary cell apoptosis, and activation of Raf-1 kinase further exaggerates this. Activation of Ras-Raf appears to result in the apoptosis of retinal capillary cells via Ras-Raf-MEK-ERK pathway. The process is executed by caspase-3, and activation of the transcriptional factor NF-*k*B also plays a role.

Our previous studies have shown that H-Ras is important in the development of diabetic retinopathy. The functional activation of H-Ras appears as one of the signaling steps involved in accelerated capillary cell apoptosis in diabetes (4, 5, 14). Activation of H-Ras and its down-stream signaling pathway in the retina and its vascular cells is under the control of superoxide (15). Activated Ras modulates divergent processes, including cell growth and apoptosis, and promotes translocation of its key receptor serine/threonine kinase, Raf-1, to the plasma membrane, leading to phosphorylation of Raf-1 (6, 16). Here we show that inhibition of Raf-1 kinase by GW5074 prevents retinal endothelial cells from increased apoptosis that they experience in high glucose conditions. This strongly suggests that the increased apoptosis of retinal capillary cells in diabetes occurs, in part, via activation of Raf-1 kinase. This is consistent with the involvement of Raf kinase in UVA-induced apoptosis of human lens epithelial cells, and the prevention of apoptosis of endothelial cells by Raf-kinase inhibitors (16, 17).

Binding of Ras to Raf initiates activation of MEK that has divergent physiological and pathological responses. Activated Raf phosphorylates MEK, which in turn, activates ERK (18, 19). Gene array studies have shown that the mRNA expression of MAP kinase increases in the retina as early as 3 days after induction of diabetes in Long-Evans rats (20). The MAP kinase pathway is implicated in the regulation of retinal vascular permeability by hepatocyte growth factor in diabetes (21), and also in glucose-induced apoptosis of retinal endothelial cells (22). Our previous studies have shown that inhibition of MAP kinase by overexpression of mitochondrial superoxide dismutase (MnSOD) also inhibits the development of retinopathy in diabetic mice (14, 23). Regulation of high-glucose-induced Raf-1 activation by its inhibitor or by its activator also regulates MAP kinase. GW5074 significantly decreased activation of p38 MAP kinase, while ZM336372 further increased its activation confirming that the Raf-mediated MAP kinase pathway is important in the development of diabetic retinopathy.

ERK is a downstream component of the Raf-MEK activated signaling pathway. Activation of MEK by Raf is followed by phosphorylation of ERK. Activated ERK results in various cellular responses, including cell immortalization and angiogenesis (6, 24). Activation of ERK is also associated with apoptosis of renal epithelial cells. Reactive oxygen species is considered as one of the mechanisms via which activation of ERK results in apoptosis (25). Our results demonstrate that inhibition of Raf-1 kinase, in addition to modulating MEK and apoptosis in retinal endothelial cell, also attenuates activation of ERK. This suggests that the Ras-Raf-MEK-ERK pathway is active in accelerating the apoptosis of retinal capillary cells in diabetic retinopathy.

Inhibition of Raf-1 kinase also prevented glucose-induced activation of the transcriptional factor, NF-*k*B, which is associated with the development of diabetic retinopathy. This implies that Raf-mediated accelerated apoptosis of retinal capillary cells in high glucose is via activation. NF-*k*B activation in the retina is an early event in the development of retinopathy. Inhibition of its activation is shown by us to prevent the development of retinopathy in diabetic rats (4, 26).

Activation of NF-*k*B modulates the inducible form of nitric oxide synthase, and this, in turn, results in increased NO production (27). Here we show that a Raf-1 kinase inhibitor prevents glucose-induced increased in NO levels in retinal endothelial cells. Reaction between superoxide and NO forms peroxynitrite which can increase DNA damage induce formation of 8-hydroxy deoxyguanosine and deplete intracellular glutathione levels (28). Peroxynitrite levels are elevated in the retina early in the pathogenesis of diabetic retinopathy and remain elevated when the histopathology characteristics of diabetic retinopathy are seen in diabetic rats (26, 29).

Glucose-induced activation of caspase-3 in the endothelial cells is inhibited by GW5074 suggesting that the Ras-Raf-MEK-ERK-mediated apoptosis of retinal capillary cells in high glucose conditions occurs via activation of caspase-3. This is consistent with our previous report showing that overexpression of dominant negative H-Ras inhibits glucose-induced activation of caspase-3 in retinal endothelial cells (5). The mechanism by which this pathway activates caspase-3 is not clear, but could include mitochondrial dysfunction. Activation of ERK is reported to regulate mitochondrial protein Bax which may result in alterations in mitochondrial permeability causing release of cytochrome c and caspase-3 activation (30, 31). In support of this concept, we have shown that retinal mitochondria are swollen and dysfunctional in diabetes, and overexpression of MnSOD prevents glucose-induced increase in capillary cell apoptosis (12, 23).

Our results demonstrate that inhibition of the Ras-Raf downstream signaling step, MAP kinase, by PD098059 results in attenuation of glucose-induced apoptosis of retinal endothelial cells, and activation of both NF-*k*B and caspase-3. These intriguing results suggest that, in addition to the upstream Ras-Raf signaling steps, the signaling molecules downstream of MAP kinase are also critical in increased apoptosis of retinal capillary cells in diabetes. Consistent with this hypothesis, activation of p38 MAP kinase is implicated in stress-induced apoptosis (32), and in the apoptosis of retinal pericytes mediated by α-dicarbonyl–modified fibronectin is postulated to contribute to the development of diabetic retinopathy (33).

In summary, Raf-1 kinase plays a significant role in the accelerated loss of retinal capillary cells in a high glucose environment. Modulation of its activity regulates apoptosis of retinal endothelial cells. This is encouraging because antisense oligonucleotides and small-molecule kinase inhibitors of Raf-1 kinase are being developed for cancer treatment (34, 35). Thus, the inhibition of Raf-1 kinase may provide an important therapeutic target in the treatment of diabetic retinopathy.
